# Novel Microwave-Assisted Method of Y_2_Ti_2_O_7_ Powder Synthesis

**DOI:** 10.3390/ma13245621

**Published:** 2020-12-09

**Authors:** Vladimir Chishkala, Serhiy Lytovchenko, Bohdan Mazilin, Edwin Gevorkyan, Vladimir Shkuropatenko, Viktor Voyevodin, Mirosław Rucki, Zbigniew Siemiątkowski, Jonas Matijošius, Agnieszka Dudziak, Jacek Caban, Artūras Kilikevičius

**Affiliations:** 1 Department of Reactor Engineering Materials and Physical Technologies, V. N. Karazin Kharkiv National University, 4 Svobody Sq., 61022 Kharkiv, Ukraine; vchishkala@ukr.net (V.C.); s.lytovchenko@karazin.ua (S.L.); mazilin@karazin.ua (B.M.); 2 Department of Quality, Standardization, Certification and Manufacturing Technology, Ukraine State University of Railway Transport, 7 Feuerbach Sq., 61010 Kharkiv, Ukraine; cermet-u@mail.com; 3 Institute of Solid State Physics, Materials Science and Technology NSC KIPT NAS of Ukraine, 1 Academichna Str., 61108 Kharkiv, Ukraine; shkuropatenko@kipt.kharkov.ua (V.S.); voyev@kipt.kharkov.ua (V.V.); 4 Faculty of Mechanical Engineering, Kazimierz Pulaski University of Technology and Humanities in Radom, ul. Stasieckiego 54, 26-600 Radom, Poland; z.siemiatkowski@uthrad.pl; 5 Institute of Mechanical Science, Vilnius Gediminas Technical University, J. Basanavičiaus g. 28, 03224 Vilnius, Lithuania; jonas.matijosius@vgtu.lt (J.M.); arturas.kilikevicius@vgtu.lt (A.K.); 6 Faculty of Production Engineering, University of Life Sciences in Lublin, Głęboka 28 Str., 20-612 Lublin, Poland; 7 Faculty of Mechanical Engineering, Lublin University of Technology, Nadbystrzycka 36, 20-618 Lublin, Poland; j.caban@pollub.pl

**Keywords:** pyrochlore, Y_2_Ti_2_O_7_, microwave irradiation, solid-phase synthesis

## Abstract

In the paper, a novel technique for highly dispersed pyrochlore Y_2_Ti_2_O_7_ is proposed. The experimental results proved that the application of microwave irradiation at a certain stage of calcination allowed synthesizing of Y_2_Ti_2_O_7_ in much shorter time, which ensured substantial energy savings. An increase up to 98 wt.% in the content of the preferred phase with a pyrochlore-type structure Y_2_Ti_2_O_7_ was obtained after 25 h of yttrium and titanium oxides calcination at a relatively low temperature of 1150 °C, while the microwave-supported process took only 9 h and provided 99 wt.% of pyrochlore. The proposed technology is suitable for industrial applications, enabling the fabrication of large industrial amounts of pyrochlore without solvent chemistry and high-energy mills. It reduced the cost of both equipment and energy and made the process more environmentally friendly. The particle size and morphology did not change significantly; therefore, the microwave-assisted method can fully replace the traditional one.

## 1. Introduction

The continuous development of materials includes a wide range of new metal alloys, such as titanium alloys [1,2], aluminum–lithium alloys [3,4], magnesium alloys [5,6], but also composite materials, e.g., ones with carbon fibers, 3D fabrics, thin layers [7,8,9,10,11], etc., and biodegradable materials [12,13]. Besides this work, the search for alternative production methods aimed to reduce energy consumption and manufacturing costs is one of the most pressing engineering problems. This progress can be seen in many areas of the economy, e.g., in the transport and the automotive industry [14,15,16,17,18], machinery industry [19,20,21,22,23], biotechnology and medicine [24,25,26], as well as the energy or electronic sector [27,28,29,30,31,32,33]. Moreover, development in the above-mentioned sectors contributes to the improvement of the quality of human life and environmental protection, which is particularly important nowadays.

One of the promising directions of the nuclear industry materials development is the production of composite materials based on ferrite-martensitic radiation-resistant steels. Such materials are oxide dispersion strengthened alloys (ODS), i.e., alloys with a steel matrix strengthened by nanosized oxide particles dispersed within it. Ferrite steels strengthened with Y–Ti–O nanoparticles have recently attracted attention as leading candidates for fission and fusion reactor components [33] because of their high tensile, creep, and radiation resistance, which is much higher than that of other alloys of iron [34,35].

Nanoparticles, for the most part, have an almost stoichiometric composition, such as Y_2_TiO_5_ and Y_2_Ti_2_O_7_ with a pyrochlore structure [36,37]. In general, the pyrochlore oxides of A_2_B_2_O_7_ type, where A is typically a rare earth ion and B is a transition metal, have a specific lattice structure, active charge, spin, and orbital degrees of freedom. As a result, they exhibit a complex interplay between geometrical frustration, electronic correlations, and spin–orbit coupling [38]. Various pyrochlores are considered good matrix materials for the isolation of actinides and other nuclear waste [39]. Pyrochlores are also refractory materials with important properties, including ionic conductivity [40], optical nonlinearity [41], high radiation resistance [42], and others. Potential applications of pyrochlores cover thermal and environmental barrier coatings [43], dielectrics with high dielectric constant [44], solid electrolytes, anodes and cathodes in solid oxide fuel cells [45], transparent ceramics [46], etc. In addition, pyrochlores have a potential application as ceramic pigments due to their high melting points, high refractive index, and the ability to absorb transition metals [47].

Even though a thermodynamic description for the TiO_2_–Y_2_O_3_ system was proposed [48], there are quite a few industrial methods of pyrochlore fabrication. The solid-phase direct synthesis of pyrochlores is one of the most common methods of forming such compounds [49], though the citric acid sol-gel method was also reported [50] and is especially useful for the fabrication of yttrium titanate thin films [51]. The manufacturing process for products made out of compounds synthesized by solid-phase reactions consists of three steps. In the first step, a homogeneous powder mixture of the desired composition and dispersion is produced. In the second one, the direct synthesis of the pyrochlore compound is carried out. Then, in the third step, the final product is formed. Depending on the particular technique, these steps may be joined together, or the sequence of individual operations may be altered. In particular, the synthesis may be performed during the formation stage of the final products. In many works, high temperatures of 1400–1500 °C and long exposure times from 12 up to 100 h or more were used to obtain powders of the synthesized pyrochlore Y_2_Ti_2_O_7_ [52]. Good results were reported after high-energy ball milling with heat treatment at 1450 °C for 12 h [53]. Other research applied a Pechini-type polymerized complex route and indicated crystallization of Y_2_Ti_2_O_7_ at 1450 °C [54]. There are also investigations where a stable Y_2_Ti_2_O_7_ phase was synthesized by the mechanical alloying method [55].

These techniques are resource and energy consuming and thus non-environmentally friendly; therefore, more economic synthesis methods are required. In [56], Y_2_Ti_2_O_7_ nanoparticles were obtained by annealing Y–Ti hydrides at 900 °C for 1 h, which were then added as an input powder to produce aluminum containing ODS steels. The Y_2_Ti_2_O_7_ particles did not decompose, but were transformed into amorphous clusters during mechanical doping. Tensile strength and uniform elongation of the ODS steels with the addition of 0.2 and 0.6 wt.% Y_2_Ti_2_O_7_ were better than that of ODS steel with 0.35 wt. % Y_2_O_3_. In another report [57], a fully crystallized pyrochlore phase was obtained from the mixture of powders Y_2_O_3_ and TiO_2_ through a heat treatment at 900 °C and mechanical milling. Pure pyrochlore Y_2_Ti_2_O_7_ was obtained also through a polymerized complex route [58], when the precursor was heat-treated in a furnace set at 750 °C in static air for 4 h, with application of mixed solution of citric acid, ethylene glycol, and yttrium and titanium ions. A microwave hydrothermal route [59] provided pyrochlore-structured Y_2_Ti_2_O_7_ on calcination at temperatures 950 and 1050 °C. Reduction of the temperature during the Y_2_Ti_2_O_7_ synthesis was achieved through the Reverse Co-precipitation (RCP) technique [60].

In the presented study, the main goal was to develop a technique for the fabrication of highly dispersed pyrochlore Y_2_Ti_2_O_7_ at possibly lower temperatures, in possibly shorter time, with no additional substances or solutions.

## 2. Materials and Methods

Thermal and thermogravimetric analyses were performed to investigate the behavior of the mixture of yttrium and titanium oxides during heating. Ceramic samples were made out of oxides of yttrium (III) Y_2_O_3_ and titanium TiO_2_. The initial powders were the industrially available ones with particle sizes 1.5–3.0 µm for TiO_2_ and 0.5–3 µm for Y_2_O_3_. First, the powders were heated separately for 90 min at 850 °C to remove the absorbed water and CO_2_. Then, the mixture of atomic percentage ratio 1:1 was prepared, which was mixed-milled in ethanol environment in a mortar made out of Al_2_O_3_. The mixing and milling procedure was performed for 2 h to obtain the required homogenization, and then the powder was compacted into sample tablets in a steel mold at a pressure of 1.2 GPa. After cold molding, the samples were subject to heat treatment.

The heat treatment of the samples was performed in two ways. In the first method, which was applied to one portion of the sample tablets, heating was carried out in a typical calcining furnace within an air environment. Here, the heat treatment at 1150 °C was performed in stages, from 7 h up to total time of 25 h.

The second method was microwave-assisted heating in a modified microwave oven at a temperature of 1300 °C for 1 and 2 h, respectively. The microwave furnace Elenberg MG-2050M type was used as the microwave radiation source. Microwave radiation parameters were 700 W, 2450 MHz. A corundum melting pot was placed inside the heat-insulating mullite-silica glass-fibrous material [61], and around it 4 cylindrical heaters with MoSi_2_ of diameter *d* = 6 mm and height *h* = 25 mm were placed. The cooling of the magnetron was carried out by forced air movement on the microwave oven housing in the area of the magnetron. Temperature control was performed using a pyrometer FLUS IR-866U (Shenzhen Flus Technology Co., Ltd., Shenzhen, China).

Phase analysis of the synthesized materials was determined by X-ray diffractometry. Diffractometry studies were performed on a DRON-4-07 X-ray diffractometer (Bourevestnik, St. Petersburg, Russia) with copper Cu-Kα radiation and a Ni-selective absorbent filter. The registration of diffraction X-rays on the samples was carried out with scintillation detector. Crystal lattice parameters were analyzed with reference to the international database and the Rietveld method [62]. An IR spectrophotometer ICS-29 (LOMO, St. Petersburg, Russia) was used to record the absorption spectra in the IR range. The spectra were recorded in the middle infrared range between 4000 and 400 cm^−1^. Differential thermal and thermogravimetric analyses (DTA/TG) were performed using an SDT Q600 V20.9 Build 20 thermo-analyzer (TA Intruments, New Castle, DE, USA) in a temperature range between 20 and 1400 °C, with a heating rate of 5 °C/min. A description of these methods can be found, for example, in [63]. The microstructure and elemental composition of the obtained samples were examined using a Scanning Electron Microscope JSM—7001F (JEOL Ltd., Tokyo, Japan).

## 3. Results and Discussion

**Figure 1:**
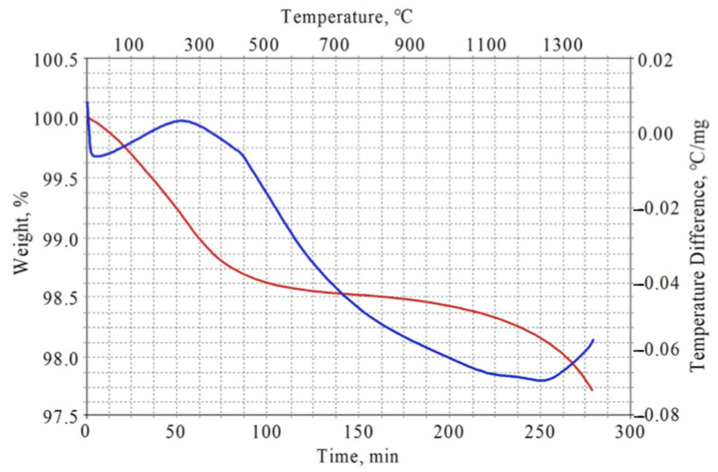
DTA (blue) and TG (red) curves of Y_2_O_3_-TiO_2_ powder mixtures.

Figure 1 presents the results of the DTA/TG analysis. It shows that the weight loss represented with the red TG curve was gradually declining with increasing temperature. This was attributed to the evaporation and removal of ethanol reminders from the heated powders. Initially, rapid weight loss remarkably slowed down after the temperature reached ca. 500 °C. An endothermic peak on the DTA curve (blue) after ca. 50 min was presumably associated with water loss. In general, the weight loss was insignificant, since it was less than 2% even at 1300 °C, but for the laboratory experiments it was important to remove all admixtures. Above temperature ca. 1100 °C, some increased weight loss was seen in the thermogravimetric (red) curve, perhaps due to the oxygen removal from titanium and yttrium oxides during pyrochlore synthesis. It can be concluded that the pyrochlore Y_2_Ti_2_O_7_ structure formation was initialized only above 1100 °C.

### 3.1. Pyrochlore Obtained in Calcining Furnace

In the first step, samples of cold molded yttrium titanate were placed in the corundum crucible and sintered in the calcining furnace at 1150 °C for 7 h. According to the initial research, a 7 h period was sufficient for the synthesis reaction. X-ray analysis of these samples revealed the presence of both initial oxides as well as the pyrochlore phase of Y_2_Ti_2_O_7_. The maximum amount of the pyrochlore phase was about 20 wt.%.

To increase the amount of pyrochlore, the samples were again mechanically powdered, mechanically homogenized, compacted, and additionally calcined at 1150 °C for 10 h. This way, the total calcining time was 17 h.

**Figure 2:**
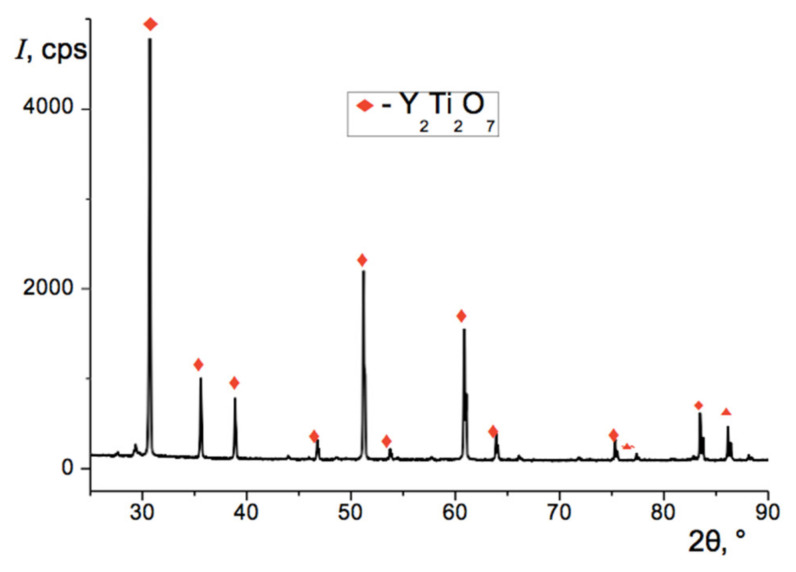
Diffraction pattern of the sample after total 17 h of calcination at 1150 °C.

According to X-ray data, after the second-stage calcination the samples became almost single- phase. The main phase was yttrium titanate Y_2_Ti_2_O_7_, its weight content in the sample was more than 96.8 wt.%, and the lattice parameter was 10.091 Å, as shown in Figure 2. In addition to the pyrochlore, traces of the initial oxides of yttrium and titanium were also found in the sample. The yttrium oxide content was ca. 0.6 wt.%, and its lattice parameter was 10.597 Å. Content of rutile TiO_2_ was close to 2.6 wt.%, and its lattice parameters were *a* = 4.591 Å and *c* = 2.999 Å.

In the third step, some of these samples were again mechanically powdered and mixed up. Then they were compacted again and calcined for another 8 h, and reached a total heat treatment time of 25 h. The additional calcination of these samples increased the Y_2_Ti_2_O_7_ content up to 98.0 wt.%, as it is presented in Table 1. This way, the desired dominant component Y_2_Ti_2_O_7_ pyrochlore structure was obtained in the sample after 25 h of calcination at 1150 °C.

**Figure 3:**
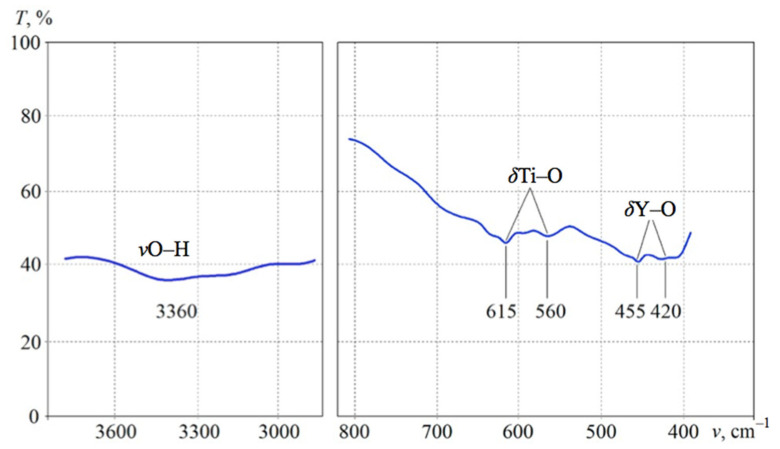
Important areas of IR absorption spectrum of the sample after 25 h of calcination at 1150 °C.

These data were confirmed by the IR analysis shown in Figure 3. The infrared spectra of the Y_2_Ti_2_O_7_ sample obtained after 25 h of calcination recorded the presence of several bands in the range between 650 and 400 cm^−1^, which was a typical property of the fluctuations of Me–O (metal-oxygen) type in cubic structures of the pyrochlore structure. The minima at 615 and 560 cm^−1^ were caused by the Ti–O deformation vibrations in the TiO_6_ octahedrons, and the minima at 455 and 420 cm^−1^ appeared due to the deformation vibrations of Y–O. In addition, there is a weak broad band in the spectrum of the valence vibrations of O–H, indicating the presence of a small number of hydroxyl groups in the sample structure.

**Figure 4:**
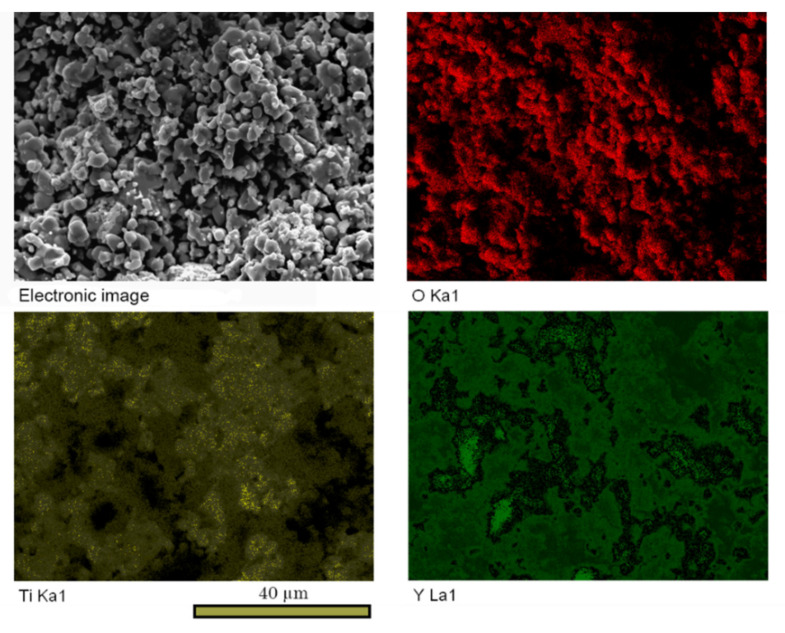
The sintered sample fracture SEM images and maps of element distribution after 25 h at 1150 °C.

Nevertheless, the content of pyrochlore after 25 h of calcination was about 98 wt.%, as it can be seen from Table 1, which indicated incompleteness of the synthesis. It was found that the rest of the sample constituted Y_2_O_3_ 0.9 wt.%, and TiO_2_ rutile ca. 1.1 wt.%. Possibly, the process was incomplete due to some errors in the weighing of the stoichiometric composition, as well as the unnoticed losses and introduction of impurities during the milling stages. Moreover, one of the reasons may be the non-uniform distribution of the powder particles Y_2_O_3_ and TiO_2_ after the first mixing procedure, which prevented them from interaction. The latter assumption seems to be confirmed by the distribution maps of the basic elements in the sample material shown in Figure 4. Even though the homogenization was performed mechanically at each of three stages, the distribution of elements in the final sample was still unsteady.

It should also be noted that after 25 h of heating, the content of Y_2_Ti_2_O_7_ mainly increased in the surface layer of the samples, but even there, some amounts of initial oxides were still present. In the core part of the sintered sample, there was a decrease in the Y_2_Ti_2_O_7_ phase content down to 93.4 wt.% accompanied with an increase of Y_2_O_3_ up to 2.7 wt.% and TiO_2_ up to 3.9 wt.%.

### 3.2. Microwave-Assisted Pyrochlore Synthesis

In the study of the effect of microwave irradiation on the synthesis of Y_2_Ti_2_O_7_ pyrochlore, several samples of powder mixtures Y_2_O_3_ and TiO_2_ were made and then treated in a modified microwave oven at different temperatures and exposure times.

Sample #1 was calcined under microwave irradiation for 1 h. The heating temperature in the microwave oven was ca. 1300 °C. According to X-ray diffraction analysis, the content of the obtained pyrochlore phase Y_2_Ti_2_O_7_ was close to 51 wt.%.

Sample #2 was first heat-treated at 1150 °C for 7 h in a conventional furnace, similarly to the first stage for samples described in the Section 3.1. After that, the sample material was mechanically powdered, compacted, and then calcined again under microwave irradiation for 1 h. As a result, Sample #2 contained a significantly larger amount of the pyrochlore structure, ca. 87 wt.%. Interestingly, assuming that after the initial stage the pyrochlore content was ca. 20 wt.%, as in position 1 Table 1, from the experiments with Sample #1 it could be expected that 1 h microwave-assisted heating would produce further Y_2_Ti_2_O_7_ with total amount close to 70 wt.%, but in fact it was much larger. This observation confirmed that the initial stage of heating in a conventional furnace had an important effect on the entire synthesis process.

Sample #3 was made in the same way as Sample #2, but the holding time in the microwave oven was prolonged up to 2 h. Significant growth of the pyrochlore structure up to 99 wt.% was registered, though it took relatively short additional time. Such a percentage was not reached even after 25 h of conventional heating. Table 2 presents the respective results of experiments.

The experimental results show that the application of microwaves after preliminary heating in a conventional furnace enabled a more effective synthesis of Y_2_Ti_2_O_7_. First of all, the process time was substantially shortened from 25 h down to a total 9 h. Consequently, less energy was consumed, making the procedure more economical and environmentally friendly. Most importantly, there was an increase in the content of Y_2_Ti_2_O_7_ up to 99 wt.%.

**Figure 5:**
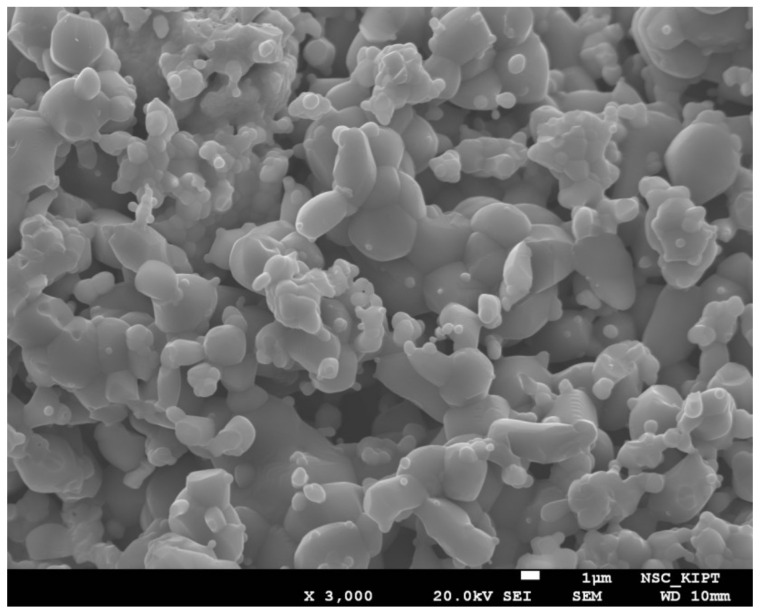
SEM image of Y_2_Ti_2_O_7_ sample after conventional heat treatment for 7 h, and 1 h microwave-assisted heating.

An analysis of the particle sizes of the synthesized pyrochlore Y_2_Ti_2_O_7_ showed that they did not significantly exceed the original size of the initial mixture powder particles. Synthesis at the temperature of 1150 °C, even at prolonged holding times, did not generate particle growth. Similarly, after additional microwave-assisted calcination, no significant increase in the particle size of the synthesized pyrochlore Y_2_Ti_2_O_7_ was observed. Figure 5 illustrates the microstructure of Sample #2 obtained after calcination for 7 h and an additional 1 h in a microwave furnace.

As it is seen in Figure 5, most of the pyrochlore particles had dimensions of a few microns. Thus, from the perspective of the obtained particles’ size and morphology, there is no significant difference between the proposed microwave-assisted method and the conventional one.

## 4. Conclusions

The experimental results demonstrated that it was possible to obtain pyrochlore Y_2_Ti_2_O_7_ powder with a content of 98.0 wt.% by solid-phase synthesis at a temperature of 1150 °C after a total 25 h. Compared to technologies involving higher temperatures, it significantly simplified the technological process and reduced production expenses.

Moreover, application of the microwaves increased further the effectiveness of Y_2_Ti_2_O_7_ pyrochlore synthesis and generated additional savings. The pyrochlore was synthesized at the same temperature of 1150 °C at a much shorter time of 9 h with higher content of 99 wt.%. Modification of the traditional technology of direct solid-phase synthesis by microwave radiation substantially shortened synthesis time and reduced energy consumption.

From a practical perspective, considering further application of the obtained powders, it was important to keep similar particle sizes in the novel procedure. It was proven that even a prolonged to 25 h exposure at temperature 1150 °C caused the particle sizes of the pyrochlore phase to only slightly exceed the dimensions of the initial powder particles. The additional microwave irradiation did not significantly affect the particle size of synthesized pyrochlore Y_2_Ti_2_O_7_.

Compared to other methods, our proposal does not provide the lowest possible synthesis temperature. However, the lower-temperature method is suitable mostly for the jointly precipitated titanium and yttrium oxides in laboratory conditions. The proposed technology, in turn, was worked out for industrial purposes and allowed for the fabrication of pyrochlore in large amounts, without solvent chemistry and high-energy mills. Introduction of the special mills can further improve the process through shortening time and decreasing temperature, but the objective of the research was to avoid special devices. The obtained pyrochlore Y_2_Ti_2_O_7_ powder can be applied in the industrial processes of steel fabrication, especially in the case of oxide dispersion strengthened alloys.

## Figures and Tables

**Table 1:** Y_2_Ti_2_O_7_ content in the samples after calcination at 1150 °C for subsequent stages of 7, 10, and 8 h.

No.	Y_2_Ti_2_O_7_ Content, wt.%	Total Calcining Time, h
1	20	7
2	96.8	17
3	98.0	25

**Table 2:** Content of Y2Ti2O7 in the samples after different heat treatments.

Sample	Y_2_Ti_2_O_7_ Content, wt.%	Total Calcining Time in the Conventional Furnace, h	Total Calcining Time in the Microwave Oven, h
#1	51	-	1
#2	87	7	1
#3	99	7	2
